# Exosome-mediated delivery of artificial circular RNAs for gene therapy of bladder cancer

**DOI:** 10.7150/jca.90620

**Published:** 2024-02-04

**Authors:** Qun Zhou, Lan Fang, Yachun Tang, Qing Wang, Xin Tang, Lexi Zhu, Na Peng, Baoyuan Wang, Wenke Song, Hao Fu

**Affiliations:** 1 The Affiliated Nanhua Hospital, Department of Urology, Hengyang Medical School, University of South China, Hengyang, 421002, Hunan, China.; 2 Department of Nursing, School of Medicine, Huainan Union University, Huainan, 232038, Anhui, China.

**Keywords:** CRISPR-Cas9, AcircRNA, β-catenin, NF-κB, Bladder Cancer

## Abstract

Bladder cancer (BCa) is one of the most common malignancies affecting men. Oncogenic transcription factors function as an important regulator in the progression of human cancer. In our study, we aimed to construct artificial circular non-coding RNAs (acircRNAs) consisting of three functional units that mimic the CRISPR-Cas system and elucidate its therapeutic role in bladder cancer. Additionally, the compare of the efficiency in regulating gene expression between acircRNA and CRISPR-dCas systems was performed. We connected the cDNA sequences of TFs aptamer and constructed a circRNA. To demonstrate the platform's practicality, β-catenin and NF-κB were chosen as functional targets, while T24 and 5637 cell lines served as test models. Real-time Quantitative PCR (qPCR), double luciferase assay and related phenotype assay were used to detect the expression of related genes and the therapeutic effect. To elucidate the functionality of acircRNAs, luciferase vectors capable of detecting β-catenin and NF-κB expression were employed to assess the inhibitory impact of acircRNA on β-catenin and NF-κB. Consequently, the optimal combination involving acircRNA-3 was determined. Next, qPCR assay was employed to assess the relative expression levels of target downstream genes following acircRNA treatment. The expression of c-myc and cyclin D1 were used to determine the function of β-catenin, while Bcl-XL and TRAF1 were used to determine that of NF-κB. The acircRNAs inhibited the β-catenin and NF-κB related signaling in BCa cells specifically. CD63-HuR fusion protein was used to loading acircRNA into exosomes. The results showed that acircRNA could inhibit the activity of the target transcription factors, and the inhibitory effect was better than that of CRIPSR-dCas9-KRAB. Furthermore, functional experiments demonstrated that the transfection of acircRNA in bladder cells resulted in decreased proliferation, enhanced apoptosis, and suppressed migration. In conclusion, our synthetic gene device exhibited anti-tumor regulatory capabilities and showed greater efficiency in tumor suppression compared to the CRISPR-dCas9-KRAB system. Therefore, our device provides a new strategy for cancer treatment and could be a useful strategy for cancer cells.

## Introduction

Bladder cancer is one of the most common malignancies affecting men. β-catenin plays a key role in overactivated Wnt-signaling pathways in a variety of cancers, including bladder cancer and prostate cancer 1. In the progression of bladder cancer, the NF-κB signaling pathway is involved in the initiation and maintenance of BCa regardless of muscle-invasive bladder cancer (MIBC). Therefore, β-catenin and NF-κB hold great potential as therapeutic targets for bladder cancer, offering promising avenues for achieving effective treatment of BCa. However, the instability of linear RNA-based RNA interference fails to effectively impede the activity of β-catenin and NF-κB in bladder cancer treatment. There is an urgent need for the development of novel targeted inhibition approaches.

CircRNA is a unique type of non-coding RNA that exists in a circular rather than a typical linear form. The circRNAs are produced by the cellular reverse splicing mechanism, which involves the fusion of the 5-terminal splicing donor site of the downstream exon with the 3-terminal splicing receptor site of the upstream exon 2, 3. There were emerging researches have showed that the structure of circRNAs is more stable and mainly regulates cell phenotype through the interaction with miRNAs 4. Functionally, however, there are much functions of circRNAs being largely unknown currently, except as molecular sponges for miRNAs 5. Accumulating evidences demonstrated that circRNAs functioned as an important factor of the miRNA regulatory networks 6. In addition, circRNAs have been proposed to function as protein sponges, and they exist in the cellular environment as RNA-protein complexes to regulate cell behaviors 7. Based on the stable structure of circRNAs, the design of circNRA-dependent intracellular regulatory devices has great application prospects. The aptamer exhibits a remarkable ability to specifically and selectively bind to diverse target substances. When the aptamer specifically binds to the target substance, it inhibits the function of that substance. However, simple nucleic acid aptamers are unstable in the intracellular environment, which greatly limits their further use. Therefore, we postulate that the inherent stability of circular RNA, in conjunction with strategic nucleic acid adaptation design, can proficiently target and suppress precise intracellular signaling molecules.

Intracellular RNA delivery mediated by nanoparticles or viral vectors is commonly used in gene therapy 8, 9. However, these nucleic acid delivery methods show different degrees of toxic side effects. Exosomes, extracellular microvesicles with diameters ranging from 30 to 150 nm, are ideal drug carriers 10, 11. Naturally occurring exosomes transport a diverse array of signaling molecules, encompassing double-stranded DNAs, RNAs, and proteins. These signals can be actively released by the exosomes into recipient cells, thereby playing a pivotal role in facilitating intercellular communication. Therefore, the synthesis of a novel exosomes encapsulating specific signaling molecules could effectively regulate the malignant phenotype of tumor cells. However, there is currently no effective strategy for delivering circRNAs to exosomes. The process of endogenous RNA sorting and encapsulation into exosomes is regulated 12, and RNA-binding proteins are involved in the sorting process of target RNA 13. In addition, synthetic exosomes were modified to achieve better targeting specificity by exosome surface protein modifications. Tetraspanin CD63 is an exosome surface marker that has been redesigned to trace and label exosomes 14, 15. According to the formation mechanism of exosomes and the principle of RNA sorting into exosomes, we hypothesized that the fusion of exosomal membrane proteins with specific RNA-binding proteins could selectively load specific RNA molecules into newly synthesized exosomes.

In our study, we fused CD63 with an RNA binding protein, which could bind the target RNA with AU rich elements (AREs) in high affinity 16. Aptamers were used to design and construct acircRNAs, which bind to β-catenin and NF-κB together to exert anti-bladder cancer function and determined their effectiveness on inhibition of BCa cells. Next, we inserted AREs into acircrNA sequences and selectively encapsulated them with exosomes, and collected these acircRNA-exosomes for the suppression of the malignant phenotype of BCa. Here, we established a novel strategy for efficiently loading circRNA cargo into exosomes, which may be promising in gene therapy for BCa.

## Materials and Methods

### Cell lines and cell transfection

The BCa cell lines (T24, 5637) used in our study were cultured in the DMEM medium (Gibco), which was supplemented with 10% fetal bovine serum (Gibco). Additionally, the normal human prostatic epithelial cell line (RWPE-1) was growth in RPMI-1640 (Gibco) medium with 10% fetal bovine serum (Gibco). All the cells were growth at the temperature of 37℃ with 5% CO2. Cell transfection was performed when cell fusion up to 70-80%. In our experiment, lipo3000 transfection kit was used for transfection.

### Construction of artificial circRNA

The sequences of β-catenin and NF-κB aptamer were derived from previous research, and these two aptamers were connected with a fixed scaffold to construct the functional unit of acircRNA 17. In addition, the pre-linear RNA of acircRNA was constructed by chemical synthesis *in vitro*.

### Dual-Luciferase reporter assay

In our study, the functions of β-catenin and NF-κB were evaluated by the activity of the dual-luciferase reporter vector, which was derived from previous studies 18.

### RNA extraction and qPCR

The total RNAs were extracted from the transfected cells by using the TRIzol reagent, and the experimental operation mainly refer to the instructions of manufacturer (Invitrogen, Grand Island, NY, United States). The cDNA required for the qPCR assay was obtained by reverse transcription of total RNA with PrimeScript RT Reagent Kit with gDNA Eraser (Takara, Japan). The qPCR assay was performed using SYBRR Premix Ex TaqTM (TaKaRa, Japan) and the experimental operation mainly referred to the instructions of manufacturer. In the data analysis of the qPCR, GAPDH was utilized to be the control of normalization. All the primers used in our work are presented in the supplemental material.

### Cell proliferation assay

In our study, CCK-8 assay was used to determine the cell proliferation of BCa cells. The specific steps of the experimental operation mainly refer to the instructions of manufacturer (TransGen, Beijing, China) and previous literature 19. In short, transfected cells were inoculated in 96-well plates and their absorbance was measured at 0h, 12h, 24h, and 36h. The data was collected from three times assay.

### Cell apoptosis assay

The level of cell apoptosis was measured by cell death detection ELISA assay. The assay was performed according to the instruction of manufacturer (Roche Applied Science). The absorbance of cytoplasmic histone-complexed DNA was quantified at 405nm by a microplate reader (Bio-Rad). The data was collected from three times assay.

### Cell migration assay

We determined the cell migration of BCa cell via the wound-healing assay. The specific steps of the experimental operation mainly refer to the previous research. Briefly, the assay was performed when the cells was up to 90% confluence in a single 6-well plate. We used a yellow pipette tip to create a clear wound in the cell layer, and the Images were taken from an optical microscope system at 0 h and 12 h. The data was collected from three times assay.

### Statistical analyses

SPSS v20.0 software (SPSS, Inc., IL, USA) was used to analyse data via Student's t tests, one-way ANOVAs, and chi-squared tests. P < 0.05 was the significance threshold.

## Results

### Design and construction of acircRNA

**Figure 1:**
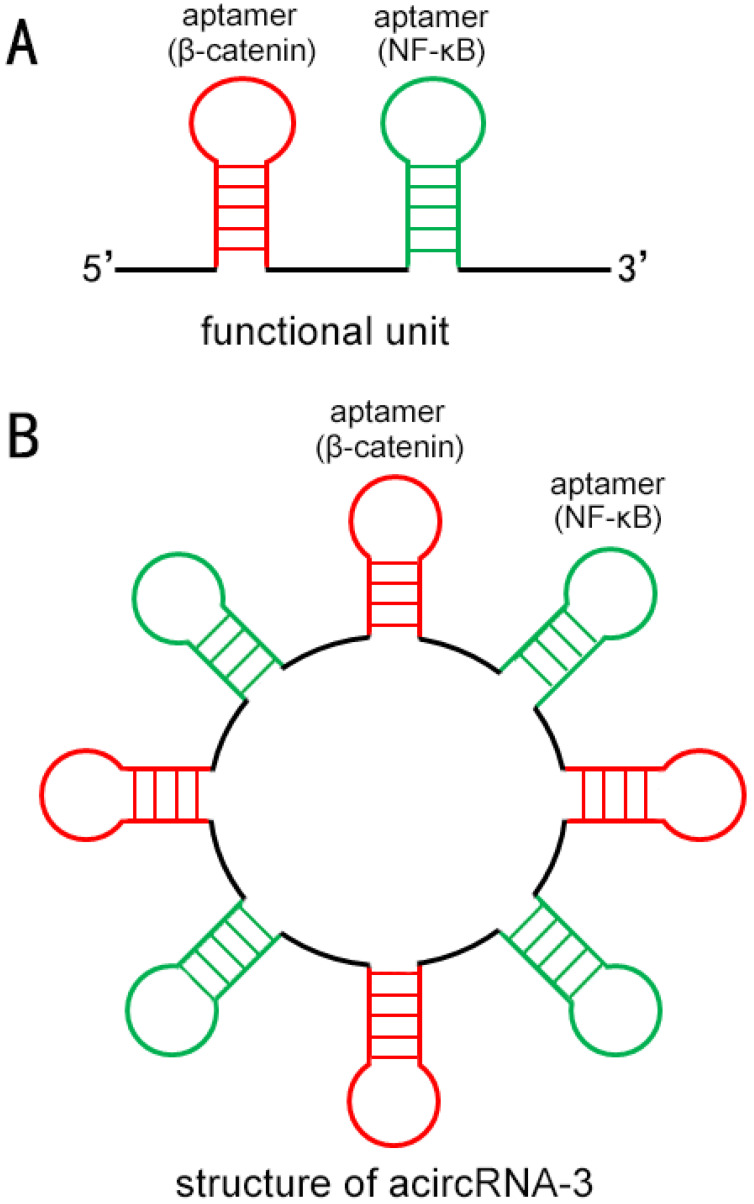
** The schematic diagram of the approximate structure of acircRNA.** (A) Each functional unit of acircRNAs was composed of one aptamer of β-catenin (Red) and one aptamer NF-κB (Green). (B) Each circular RNA consisted of three functional units.

To construct acircRNAs that specifically bind target proteins, we preliminarily designed short RNAs that can be efficiently cyclized by endogenous RNA ligase. Aptamers were used to bind specifically to target molecules β-catenin and NF-κB in our work, and each functional unit of acircRNAs was composed of one aptamer of β-catenin and one aptamer NF-κB (Figure 1A). To make the single linear pre-sequences of aptamers smoothly fold into specific functional structures, we refer to the design of previous studies 17. To cyclize the linear pre-short RNA molecule, we supplemented reverse complementary introns at both ends of the sequence (Figure 1B).

The acircRNA sponged and inhibited β-catenin and NF-κB in BCa cells specifically.

**Figure 2:**
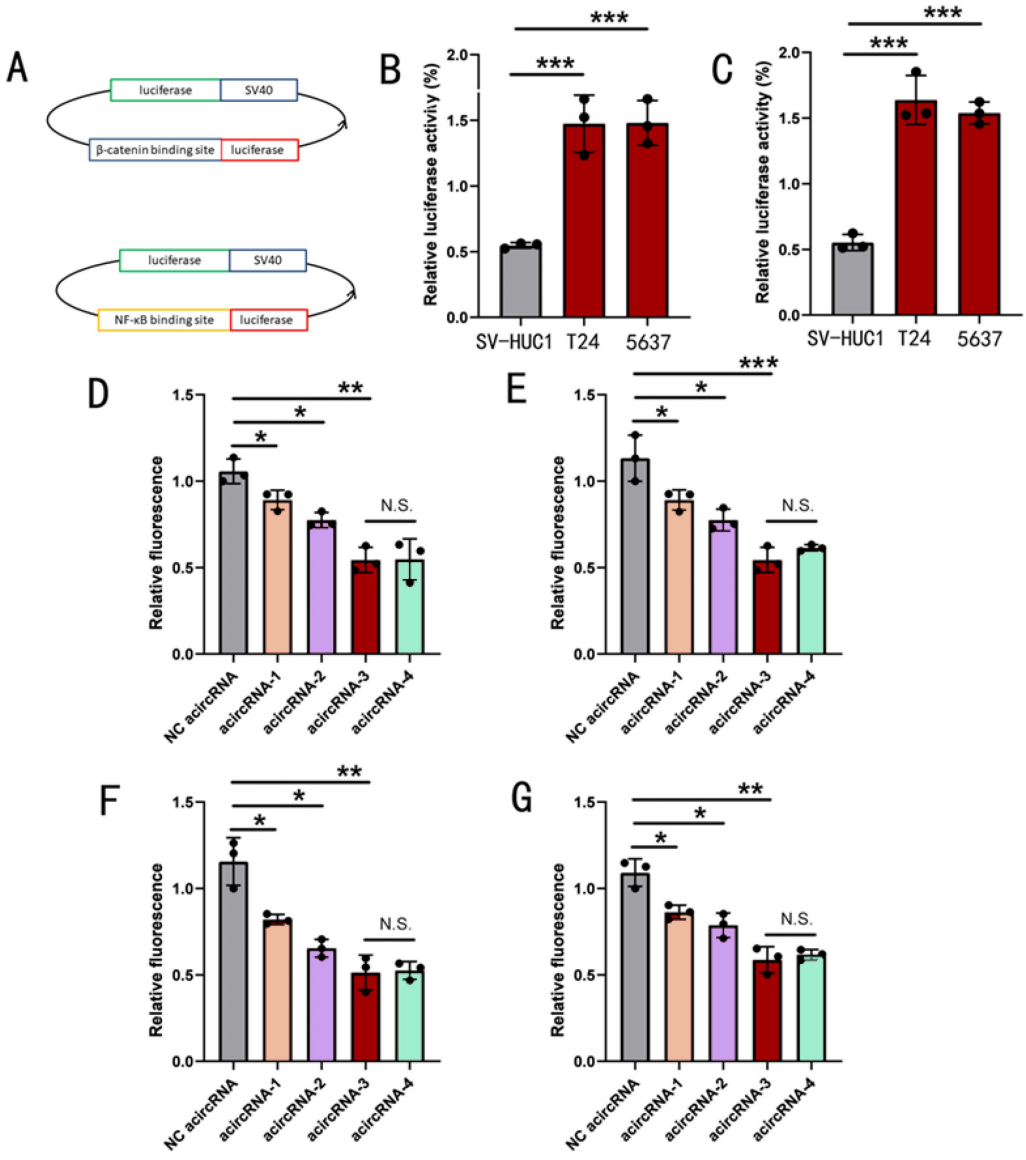
** The effect of acircRNAs inhibiting β-catenin and NF-κB in bladder cancer cells.** (A) The description of working mechanism about transcript factor-related luciferase reporters. (B) The related expression of β-catenin detected using ELISA assay in SV-HUC1, T24 and 5637 cells. (C) The related expression of NF-κB detected using ELISA assay in SV-HUC1, T24 and 5637 cells. (D) Inhibition of β-catenin by different combination strategies of β-catenin aptamers in T24 cells. (E) Inhibition of β-catenin by different combination strategies of β-catenin aptamers in 5637 cells. (F) Inhibition of NF-κB by different combination strategies of NF-κB aptamers in T24 cells. (G) Inhibition of NF-κB by different combination strategies of NF-κB aptamers in 5637 cells. Results are shown as mean±SD. ***P < 0.001 compared with SV-HUC1. **P < 0.01 compared with SV-HUC1. *P < 0.05 compared with SV-HUC1.

To determine the function of acircRNAs, the luciferase vectors capable of sensing the expression of β-catenin and NF-κB were used to detect the inhibitory effect of acircRNA on β-catenin and NF-κB (luci-β-catenin and luci- NF-κB) (Figure 2A). To determine the detection efficiency of luci-β-catenin and luci-NF-κB, we first measured the protein expression of β-catenin and NF-κB in T24, 5637 and SV-HUC1 using ELISA assay. We found that both β-catenin and NF-κB showed varying degrees of upregulation in T24 and 5637, relative to SV-HUC1 (Figure 2B and 2C). Next, the luci-β-catenin and luci-NF-κB were transfected into the BCa cells and the normal bladder epithelium cells and the results showed that the expression ratio of relative fluorescence intensity was consistent with ELISA results, which demonstrated that luci-β-catenin and luci-NF-κB could sense the expression level of β-catenin and NF-κB.

We co-transfected acircRNAs containing different numbers of functional units (from one to five units) with luci-β-catenin and luci-NF-κB into BCa cell lines, respectively, to detect the inhibitory effect of circRNAs with different construction strategies (from acircRNA-1 to acircRNA-4), and to hope for the optimal circRNAs construction scheme (Figure 2). As shown in figure 2, acircRNA-3 showed the strongest inhibitory effect on both β-catenin and NF-κB, while acircRNA-1 showed almost no inhibitory effect on β-catenin and NF-κB. In addition, acircRNA-3 and acircRNA-4 showed no significant difference in inhibition of β-catenin and NF-κB (Figure 2). We concluded that acircRNA-3 exhibited the best inhibition efficiency for β-catenin and NF-κB. Therefore, we selected the acircRNA-3 for the further experiments.

The acircRNAs inhibited the β-catenin and NF-κB related signaling in BCa cells specifically.

Through luciferase reporter assay, we found that acircRNAs could obviously bind to β-catenin and NF-κB specifically. However, it was still not clear that acircRNAs could block β-catenin and NF-κB from functioning through interactions with downstream signaling. In other words, we need to prove that acircRNAs specifically bind to β-catenin and NF-κB and inhibit their related signaling pathways.

**Figure 3:**
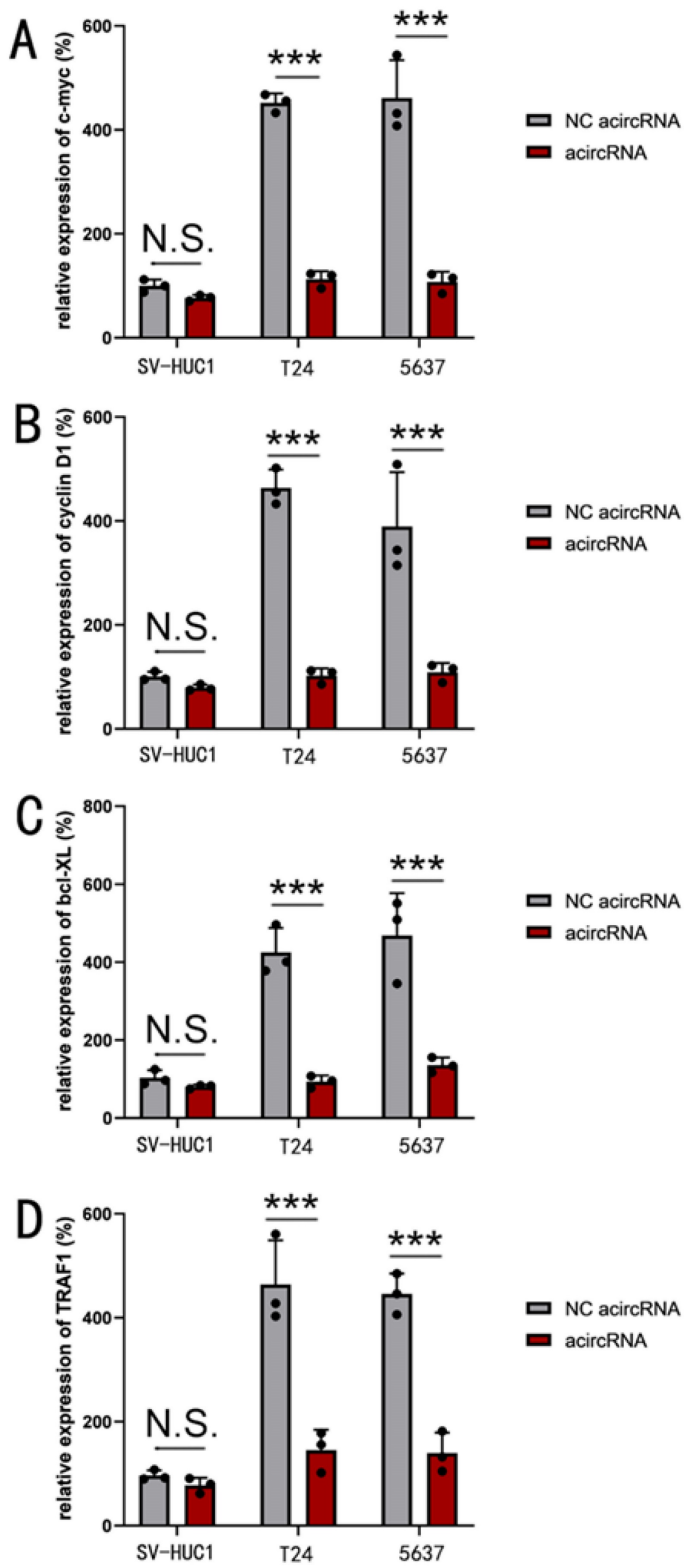
** The acircRNA inhibited the mRNA expression of various downstream genes of β-catenin and NF-κB in bladder cancer cells.** (A) The acircRNA suppressed the expression of c-myc mRNA in bladder cancer cells (SV-HUC1, T24 and 5637). (B) The acircRNA suppressed the expression of cyclin D1 mRNA in bladder cancer cells (SV-HUC1, T24 and 5637). (C) The acircRNA suppressed the expression of bcl-XL mRNA in bladder cancer cells (SV-HUC1, T24 and 5637). (D) The acircRNA suppressed the expression of TRAF1 mRNA in bladder cancer cells (SV-HUC1, T24 and 5637). Results are shown as mean±SD. ***P < 0.001 compared with SV-HUC1. **P < 0.01 compared with SV-HUC1. *P < 0.05 compared with SV-HUC1.

To further investigate the mechanism of acircRNAs, qPCR assay was utilized to evaluate the relative expression levels of target downstream genes subsequent to acircRNA treatment. The expression of c-myc and cyclin D1 were used to determine the function of β-catenin, while Bcl-XL and TRAF1 were used to determine that of NF-κB. We found that acircRNA showed significant inhibitory effect on both c-myc and cyclin D1, as well as Bcl-XL and TRAF1 (Figure 3).

### Efficient loading of acircRNAs into exosomes by CD63-HuR fusion protein

**Figure 4:**
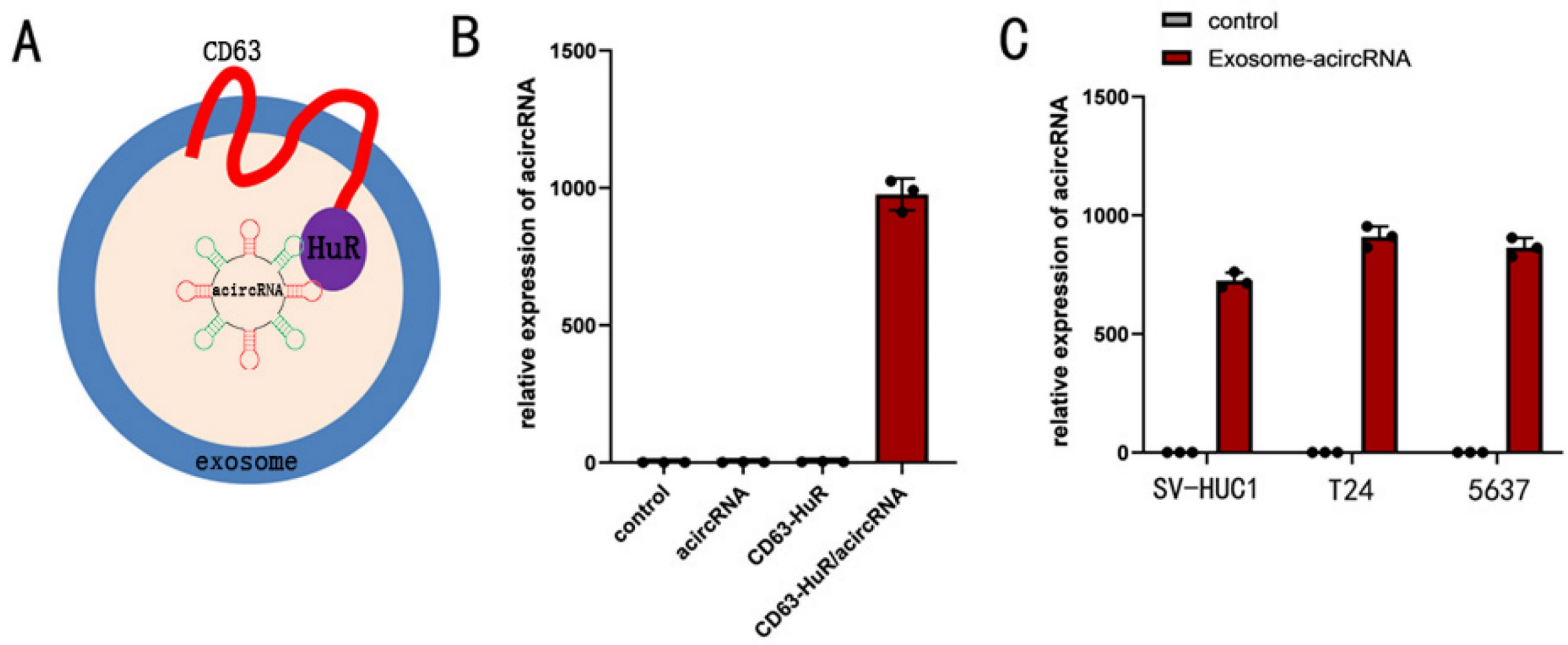
** Efficient acircRNA encapsulation into the CD63-HuR fusion protein functionalized exosomes.** (A) Schematic diagram of the structure of the functional exosome of CD63-HuR fusion protein. (B) Expression of acircRNA in HEK293T cells measured as indicated. (C) Expression of acircRNA in exosomes derived from HEK293T cells transfected into SV-HUC1, T24 and 5637 cells as indicated. Results are shown as mean±SD. ***P < 0.001 compared with control. **P < 0.01 compared with control. *P < 0.05 compared with control.

Exosomes have been widely recognized as drug delivery vehicles. Given that endogenous RNA is selectively sorted into exosomes by RNA-binding proteins, we asked whether exosomes could be re-engineered to select target RNA molecules for entry into exosomes 11, 20. To this end, we used RNA binding protein HuR to re-engineer tetraspanin CD63 and proved its feasibility. In our study, RNA binding protein HuR was selected to fuse to the C-terminus of CD63 (Figure 4). The 293t cells were co-transfected with 100nM acircRNA which including AREs and CD63-HuR expressing vector to produce the special exosomes encapsulated acircRNA. In addition, the acircRNA without AREs were co-transfected into 293t cells with CD63-HuR expressing vector to serve as control group. The results of qPCR assay revealed that acircRNA was abundant in the supernatant of acircRNA-AREs/CD63-HuR co-transfected cells, whereas it was almost absent in the control group (Figure 4). Next, the exosomes encapsulated with acircRNAs were extracted from the cell supernatant and transfected into BCa cell. The results showed that exosomes efficiently delivered acircRNAs into BCa cell (Figure 4).

### The exosome delivery acircRNA showed higher inhibitory effect of β-catenin and NF-κB than CRISPR-Cas9 system

**Figure 5:**
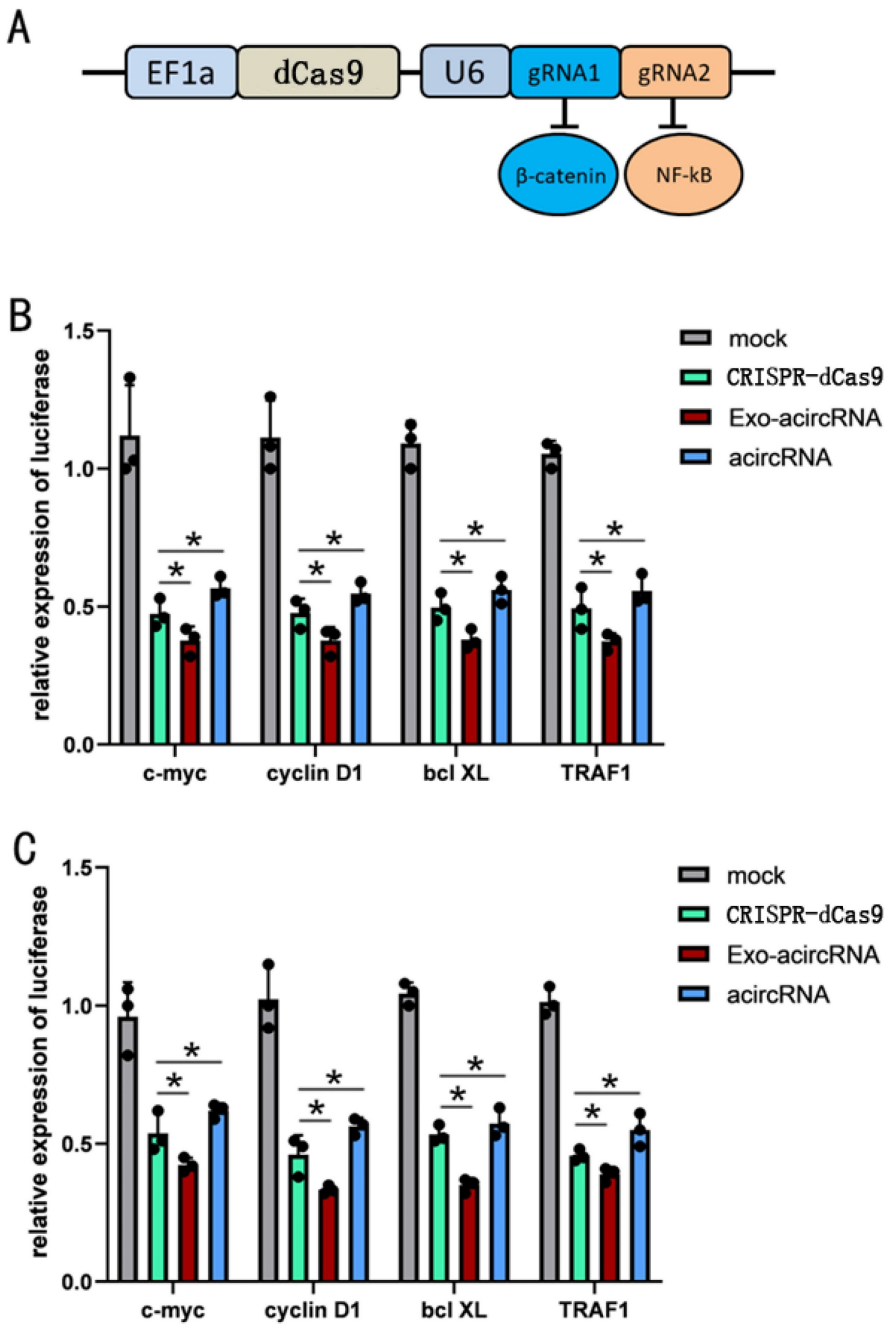
** The inhibitory effect of Exo-acircRNA was better than CRISPR-Cas9 and acircRNA.** (A) The construction of Cas9 and the targets of gRNA. (B) The qRT-PCR assay analysed the expression levels of c-myc, cyclin D1, bcl XL and TRAF1 in the BCa cell line T24 after transfected with the acircRNA and CRISPR-Cas9 compared with exo-acircRNA. (C) The qRT-PCR assay analysed the expression levels of c-myc, cyclin D1, bcl XL and TRAF1 in the BCa cell line 5637 after transfected with the acircRNA and CRISPR-Cas9 compared with exo-acircRNA. ***P < 0.001 compared with control. **P < 0.01 compared with control. *P < 0.05 compared with control.

As a robust gene editing tool, CRISPR-Cas9 has completely subverted all aspects of biological research including cancer. Here, we wanted to compare the inhibitory effects of acircRNAs and CRISPR-Cas9. Fusion of Cas9 protein without nuclease activity (dCas9) with krüppel-associated box (KRAB) repressor (dCas9-KRAB) could inhibit the expression of target genes. We targeted CRISPR-dCas9-KRAB to the promoters of β-catenin and NF-κB to inhibit them expression (Figure 5). Then we compared the CRISPR-dCas9-KRAB system and acircRNAs for inhibiting target genes.

We found that both CRISPR-dCas9-KRAB and acircRNAs significantly inhibited β-catenin and NF-κB, but interestingly, acircRNAs showed a better inhibitory effect than CRISPR-dCas9-KRAB in BCa cell lines (Figure 5).

### The exosome deliver acircRNAs inhibited the malignant phenotype of BCa *in vitro*

**Figure 6:**
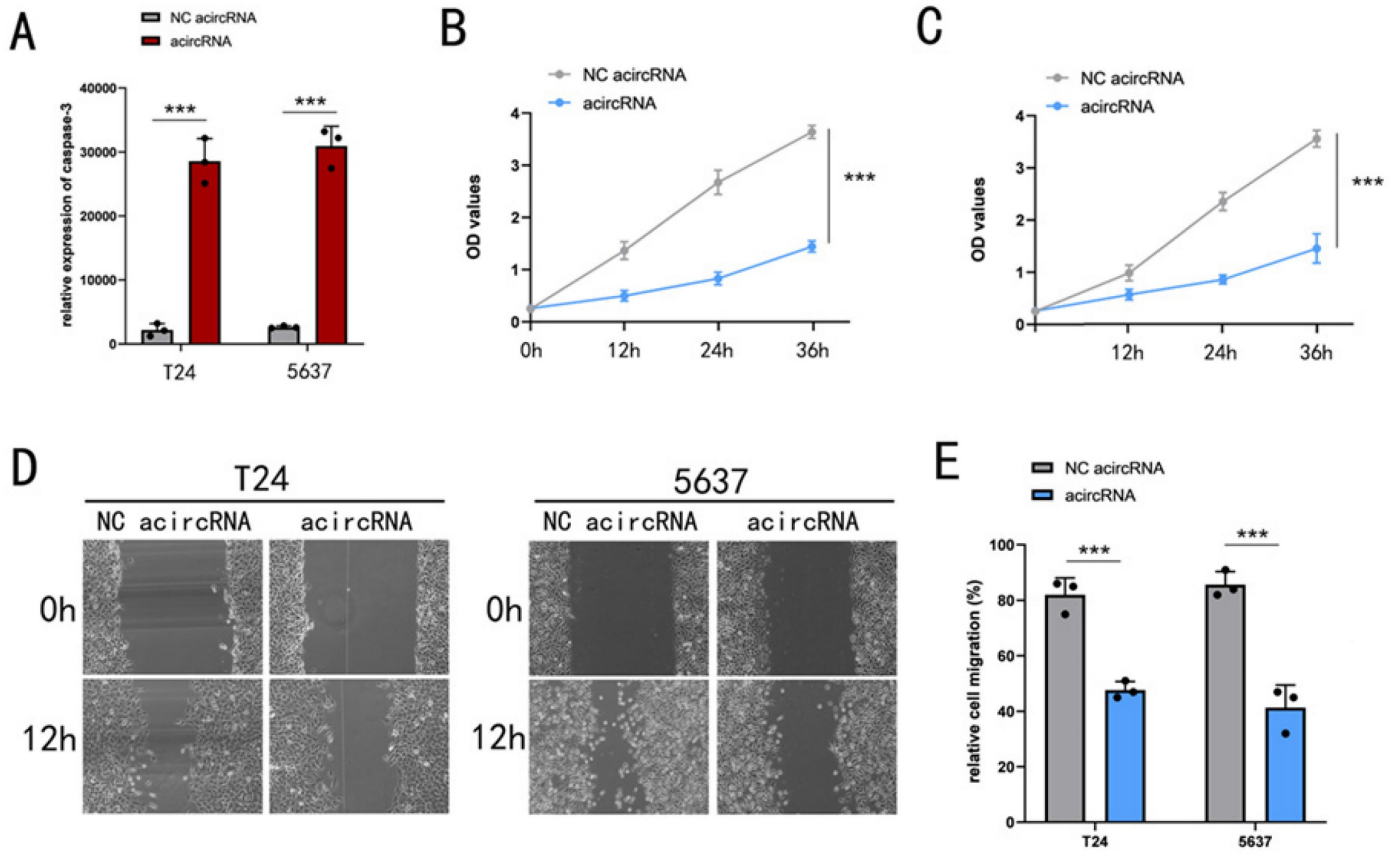
** The significant inhibitory effect of exo-circRNA on bladder cancer cells.** (A) The ELISA assay showed the relative expression of caspases-3 in BCa cell lines with and without exo-acircRNA. (B) The CCK-8 assay determined the cell proliferation in T24 cell lines with and without exo-acircRNA. (C) The CCK-8 assay determined the cell proliferation in 5637 cell lines with and without exo-acircRNA. (D) The relative rate of cell migration was determined in T24 and 5637 cells with or without exo-circRNAs using the wound-healing assay. (E) Quantification of the relative rate of cell migration was determined in T24 and 5637 cells with or without exo-circRNAs using the wound-healing assay. ***P < 0.001 compared with control. **P < 0.01 compared with control. *P < 0.05 compared with control.

Previously, we had demonstrated that acircRNAs were used to interfere with β-catenin and NF-κB associated oncogenic signaling pathways. Firstly, we investigated the inhibition of exo-acircRNAs on malignant phenotype of BCa cells. ELISA assay was performed to evaluate acircRNAs induced apoptosis levels. Apoptosis levels of BCa cell lines (T24 and 5637) that transfected with acircRNAs were determined by ELISA (Figure 6). Based on these experimental results, we concluded that the exo-acircRNAs we designed could effectively induce BCa cell apoptosis. Previous studies have reported that β-catenin inhibits apoptosis by inhibiting the effects of cleaved PARP-apoptosis protein. Therefore, exo-acircRNAs induced BCa cell apoptosis by suppressing β-catenin related pathways.

Next, we investigated whether exo-acircRNA could effectively inhibit cell proliferation in BCa cell lines (T24 and 5637). C-myc and Cyclin D1 were well-known cell cycle regulators and served as carcinogenic factors function on promoting cell proliferation. In previous experimental results, we have shown that exo-acircRNA does indeed down-regulate the expression of c-myc and Cyclin D1. In our study, cell proliferation of BCa cell lines T24 and 5637 were determined by CCK-8 assay (Figure 6). Through comparison, we found that exo-acircRNA could effectively inhibit cell proliferation of BCa cells.

Finally, we sought to clarify whether exo-acircRNA functions as a cell migration inhibitor in BCa cells. It had been reported that targeted β-catenin, as well as NF-κB, induce cancer cell migration and invasion by inducing epithelial-mesenchymal transformation (EMT). We hypothesized that exo-acircRNA inhibited BCa cell migration by inhibiting EMT. In addition, we found that cell migration of BCa cells was significantly reduced in the presence of exo-acircRNA (Figure 6).

## Discussion

Worldwide, the incidence of bladder cancer is one of among the highest among all malignancies 21. BCa is a malignancy characterized by a significantly elevated recurrence rate within the urinary system 22. After surgical treatment, patients still have a high probability of recurrent bladder cancer, and the recurrence of tumors has a trend of increasing malignancy. With the in-depth research on the pathogenesis of bladder cancer and the identification of bladder cancer-related genes, the rise of gene therapy provides a new thinking direction for the treatment of bladder cancer 23.

Aptamers needed to be screened *in vitro* with a specific series of nucleotide sequences, which can bind to a specific ligand with high affinity and strong specificity 24. In theory, any small molecule has a specific aptamer. We utilized the relationship between aptamers and ligands to construct the acircRNAs that specifically silenced carcinogenic signaling and inhibit BCa cells. β-catenin and NF-κB are well-known cancer-related factors, and the pathways involved in them regulate tumor progression, and both of them play key roles in these pathways 25, 26. In our study, we linked the aptamers of β-catenin and NF-κB together to construct the functional unit of acircRNA. The anticancer acircRNA molecule here was constructed by effectively binding the aptamers sequence of the oncogenic transcription factor. The acircRNA constructed by us inhibits the malignant phenotype of BCa cells by specifically binding to oncogenic factors. The results showed that the nucleic acid molecule constructed in the form of acircRNA could effectively inhibit the malignant phenotype of BCa cells. Meanwhile, the inhibitory effect of acircRNA was superior to that of CRISPR-dCas9-KRAB system. This strategy to inhibit the progression of BCa may also provide new ideas for the treatment of other malignancies.

However, there were still some deficiencies in our work, which will be investigated in further work. First, our work focused on the *in vitro* level and could not prove that acircRNA is still effective in complex bodies. The key is still the problem of gene delivery, in the future, we plan to use exosome or viral vectors to carry these acircRNAs devices into tissue cells. At present, we do not have sufficient evidence to prove the effectiveness of acircRNA *in vivo*, which requires more evidence to prove the clinical value of this strategy. Secondly, in our work, we mainly used transfection method to introduce acircRNAs into cancer cells. However, the delivery efficiency of this method is not high, especially *in vivo*. Therefore, the development of biological vectors that can efficiently transport acircRNA into cells is the focus of our further work. In conclusion, we successfully constructed artificial circRNAs that inhibited different tumor factors to effectively inhibit bladder cancer, which may be a potentially effective treatment strategy for advanced bladder cancer.

## Supplementary Material

Supplementary table.

## Abbreviations

CRISPR-Cas: clustered regularly interspaced palindromic repeats-CRISPR-associated proteins
KRAB: Krüppel-associated box
BCa: bladder cancer
qRT-PCR: quantitative Real Time PCR
AcircRNA: Artificial circular Ribonucleic Acid
CircRNA: circular Ribonucleic Acid
NF-κB: nuclear factor kappa-B
MIBC: Muscle-invasive bladder cancer
AREs: The adenine and uridine-rich elements
