# Supplementary material for: Exosome-mediated delivery of artificial circular RNAs for gene therapy of bladder cancer
Source: J Cancer. 2024 Feb 4;15(6):1770–8. doi: 10.7150/jca.90620 (PMC10869980; doi:10.7150/jca.90620)
Supplement: Supplementary file 1 — Supplementary table. [file jcav15p1770s1.pdf]

Supplementary table 1: Sequence of primers used in this study

name	relative sequences (5′-3′)
MYC	F: GGCTCCTGGCAAAAGGTCA
	R: CTGCGTAGTTGTGCTGATGT
Cyclin D1	F: GCTGCGAAGTGGAAACCATC
	R: CCTCCTTCTGCACACATTTGAA
TRAF1	F: TCCTGTGGAAGATCACCAATGT
	R: GCAGGCACAACTTGTAGCC
BclXL	F: GAGCTGGTGGTTGACTTTCTC
	R: TCCATCTCCGATTCAGTCCCT
acircRNA	F: GGTTCGGTCATGTGTGTGG
	R: CGGACCAGGTACTCATGCATCC
